# Univariate and multivariate analysis of the styrofoam fixation device on patient setup errors in radiotherapy

**DOI:** 10.1002/acm2.70181

**Published:** 2025-07-15

**Authors:** Jun Chen, Yi Guo, Changsheng Wang, Simin Lin, Linglong Shao, Linzhen Lan, Feibao Guo

**Affiliations:** ^1^ Department of Radiotherapy Cancer Center The First Affiliated Hospital, Fujian Medical University Fuzhou China; ^2^ Department of Radiotherapy National Regional Medical Center Binhai Campus of the First Affiliated Hospital Fujian Medical University Fuzhou China; ^3^ Key Laboratory of Radiation Biology of Fujian Higher Education Institutions the First Affiliated Hospital Fujian Medical University Fuzhou China; ^4^ Department of Radiology The First Affiliated Hospital, Fujian Medical University Fuzhou China; ^5^ Department of Radiology National Regional Medical Center Binhai Campus of the First Affiliated Hospital Fujian Medical University Fuzhou China

**Keywords:** radiotherapy, setup errors, Styrofoam

## Abstract

**Background:**

Styrofoam is a patient‐specific immobilization device in radiotherapy; most previous studies about the impact of styrofoam on setup errors have only analyzed a single tumor type, and have not considered the influence of patient's physical condition on the setup errors of styrofoam.

**Purpose:**

This study aims to evaluate the impact of styrofoam device on setup errors in radiotherapy and explore which patient population is more suitable for styrofoam immobilization.

**Methods:**

Univariate and multivariate analyses were conducted to compare the setup errors between the experimental group (styrofoam combined with thermoplastic mask) and the control group (thermoplastic mask alone). All cases were categorized based on tumor location into head and neck, thorax, abdomen, and limb cases, with age, gender, surgical history, educational level, and body mass index (BMI) serving as variables in the multivariate analysis. Intragroup analysis was also performed.

**Results:**

For all included cases, the experimental group had median setup errors of 1.20 , 2.00 , and 1.30 mm in the vertical, longitudinal, and lateral directions, respectively. In contrast, the control group had median setup errors of 1.50 , 2.00 , and 1.85 mm in the same respective directions. Notably, the experimental group demonstrated statistically significant reductions in average setup errors in the longitudinal direction (2.00  vs. 2.87 mm, *p *< 0.01) and lateral direction (1.90  vs. 2.24 mm, *p* < 0.01) compared to the control group. The intragroup analysis results indicated that factors such as age, gender, surgical history, and educational level had no significant impact on the setup errors in the experimental group.

**Conclusion:**

The styrofoam fixation device for patient immobilization can effectively reduce setup errors in both the longitudinal and lateral directions, and the styrofoam fixation device is suitable for most people.

## 1 INTRODUCTION

Patient immobilization is crucial in radiotherapy, as it restricts patient's movement during treatment and reduces setup errors, enhancing radiotherapy precision.^1^ The implementation of patient immobilization must be tailored to the patient's tumor type and physical condition. Catona and Marcu have shown that a reduction of 200 cGy in the clinical target volume (CTV) dose, which is 4% of a prescribed dose of 5000 cGy, caused by patient immobilization deviation, could potentially decrease the tumor control rate by 5%–8%.^2^ The selection of immobilization devices requires minimal impact on the dose distribution, effective immobilization, high reproducibility, and patient comfort. Thermoplastic masks, commonly used for patient immobilization, may shrink and deform over time, affecting patient setup errors, especially in prolonged treatment periods.^3^ Shafaii‐Erfani et al. reported setup errors of 1.1 , 2.0 , and 2.3 mm in lateral, longitudinal, and vertical directions for thermoplastic masks with headrests.^4^ M. Mattke et al. found an average setup error of 2.28 mm in the longitudinal direction for whole brain radiotherapy patients using head and neck masks, necessitating movement restrictions.^5^ Other studies indicate that head thermoplastic masks with headrests may not conform to every patient's neck curvature, potentially causing internal deformation as patients adjust their necks for comfort.^6^, ^7^ These studies highlight the limitations and challenges of thermoplastic masks in patient immobilization.

Styrofoam is a patient‐specific immobilization device that requires minimal patient cooperation. Its advantages include the ability to actively mold according to the body's structure, automatically filling gaps in various parts of the body to achieve individualized patient immobilization. The styrofoam consists of isocyanate (solution A), composite polyether polyol (solution B), and a polyethylene bag. The styrofoam immobilization process is standardized across all patients, with only the polyethylene bag varying by tumor location. To use the styrofoam, mix the specified amounts of solution A and B thoroughly, then pour the mixture into the polyethylene bag. Lima et al. found that the combined use of styrofoam and thermoplastic masks helps to immobilize the mandible in patients with head and neck tumors.^8^ Raphael et al. also applied styrofoam in abdominal conformal radiotherapy.^9^ Other study indicated that styrofoam has been used in Gamma Knife treatments.^10^ These studies collectively suggest that styrofoam is an effective tool for patient immobilization in radiotherapy, offering value in improving the radiotherapy precision and enhancing patient comfort. However, previous studies on styrofoam's impact on setup errors often focused on a single tumor type and didn't consider its applicability to different tumor types. Also, using univariate analysis to compare setup errors between styrofoam and other devices ignores other potential variables, potentially leading to inaccurate results. Thus, considering factors like the patient's physical condition and conducting multivariate analysis is necessary.

The primary contribution of this study lies in its multivariate analysis of setup errors. By considering factors such as immobilization devices, gender, age, educational level, surgical history, body mass index (BMI), and treatment position, this analysis aims to explore the applicability of styrofoam immobilization across different tumor types. In addition, intragroup analyses were also performed on both the styrofoam and thermoplastic mask groups. These analyses seek to identify which patient population may be more suitable for styrofoam immobilization.

## 2 METHODS

### 2.1 Patient data

**FIGURE 1:**
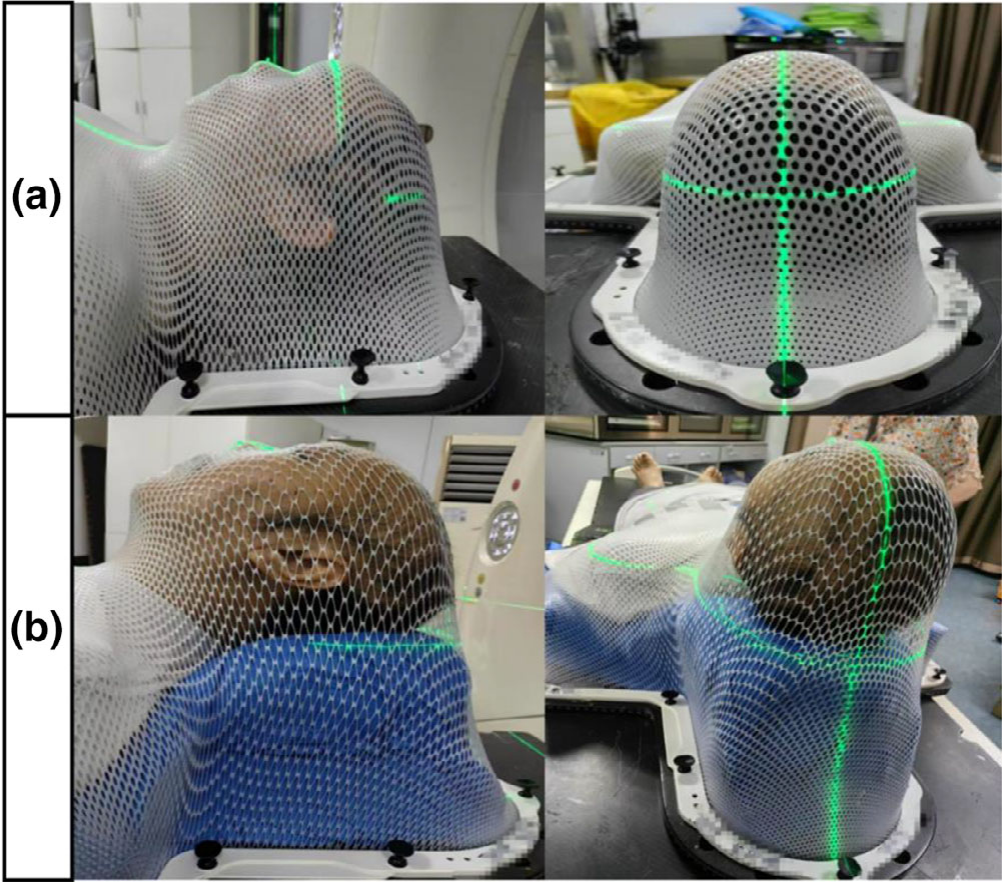
Thermoplastic mask fixation (a) and Styrofoam combined thermoplastic mask fixation (b). ns: *p* > 0.05, *p < 0.05, **p < 0.01, ***p < 0.001.

A total of 273 patients, including those with tumors of the head and neck, thorax, abdomen, and limb, who underwent radiotherapy from January 2018 to December 2023 were collected. All patients were divided into two groups: the control group used thermoplastic masks for patient immobilization, including head‐neck‐shoulder thermoplastic masks (Klarity, R460ST, Guangzhou, China), as shown in Figure 1a, and body thermoplastic masks (Klarity, R320‐S24A, Guangzhou, China). The experimental group used styrofoam (ForRad, Guangzhou, China) combined with thermoplastic masks for patient immobilization, as shown in Figure 1b. The styrofoam immobilization procedure is as follows: Mixed the solution A and B about 10 s, poured the mixture into a polyethylene bag. Placed the patient on top of the bag. The operator then moved the bag to ensure the mixture fully contacted and wrapped around the body, minimizing gaps. After approximately 10 min, the mixture will foam, expand, and solidify. Although all cases in this study were randomly selected, styrofoam immobilization was only used for patients with kyphosis in head and neck cases. This is mainly attributed to the limited movement in the head and neck region, where thermoplastic mask already meet clinical immobilization requirements. For patients with kyphosis, whose cervical physiological curvature prevents exclusive thermoplastic mask immobilization, a combination of styrofoam and thermoplastic mask was used. After patient immobilization, computed tomography (CT) scans were performed using a large‐bore CT simulator (Siemens, SOMATOM, Germany), with a slice thickness of 3 mm and a resolution of 1 mm × 1 mm. The general clinical data of the two groups are shown in Table 1.

**TABLE 1:** The general clinical data of the control group and the experimental group.

Data	Experimental group	Control group
Age	6∼86 (median 57)	14∼84 (median 56)
Gender		
Male	81	67
Female	54	71
Educational level		
Below junior high school	64	66
Junior high school and above	71	72
Surgical history		
No	57	76
Yes	78	62
BMI	22.73 ± 3.26	23.33 ± 3.65
Position		
Head and neck	34	38
Thorax	31	32
Abdomen	41	44
Limb	29	24

### 2.2 Acquisition of patient setup errors

Patient setup was performed by two experienced technicians during the initial treatment. An external laser positioning system is used to align the patient's skin markings, which are manually drawn by operators based on the position of the external laser lines on the patient's body before CT scanning. Cone beam CT (CBCT) images were acquired by a low‐dose radiation scan on the Infinity linac (Elekta, Sweden) with the following parameters: scan voltage of 110 kV, current of 20 mA, slice thickness of 2.5 mm, and a reconstruction volume of 384 × 384. The patient's CBCT were automatically registered with the simulated CT using either grayscale or bony alignment. After automatic registration, manual adjustments were made to determine the setup errors in the vertical, longitudinal, and lateral directions.

### 2.3 Analysis of the impact of styrofoam on setup errors

#### 2.3.1 Intergroup analysis

(1) Univariate analysis was used to explore the impact of styrofoam on the initial setup errors in the vertical, longitudinal, and lateral directions between the control group and the experimental group. The analysis was stratified by tumor location, with separate comparisons for head and neck, thorax, abdomen, and limb, as well as all cases combined. Since some setup error data did not meet normal distribution criteria, the Mann‐Whitney *U* test was employed. (2) Multivariate analysis. As shown in Table 2, considering that age, gender, educational level, surgical history, and BMI may influence patient setup errors, these factors were included in the multivariate analysis of setup errors for all cases, head and neck cases, thorax cases, abdomen cases and limb cases respectively. A linear regression was adopted in the multivariate analysis. (3) Anatomical deviations. Since the goal of image registration between CT and CBCT is to align anatomical structures, and setup errors do not directly indicate anatomical differences in regions of interest (ROI), the dice similarity coefficient (DSC) and average symmetric surface distance (ASSD) of ROIs on CT and CBCT were assessed.^11^ A senior physician delineated the ROIs on both CT and CBCT. For head and neck cases, the ROIs included nasopharynx gross tumor volume (GTV‐nx), node gross tumor volume (GTV‐nd), clinical target volume 1 (CTV1), clinical target volume 2 (CTV2), brain stem, spinal cord, lens, optical nerve, optical chiasm, parotid, thyroid. For thoracic cases, the ROIs included planning target volume (PTV), lung, spinal cord. For abdominal cases, the ROIs included GTV, CTV, intestine tract, bladder, femoral head.

**TABLE 2:** Variables in multivariate analysis.

Variable name	Variable type	Class
Independent variables		
Fixation device	Categorical	0: Styrofoam and thermoplastic mask 1: Thermoplastic mask
Gender	Categorical	0: Male 1: Female
Age	Continuous	/
Educational level	Categorical	0: Below junior high school 1: Junior high school and above
Surgical history	Categorical	0: No 1: Yes
BMI	Continuous	/
Dependent variable		
Setup errors	Continuous	/

#### 2.3.2 Intragroup analysis

To further determine which patient group might benefit more from styrofoam immobilization, a multivariate analysis of setup errors was performed on both the experimental and control groups. This analysis incorporated six variables: age, gender, educational level, surgical history, BMI, and tumor location (with head and neck cases serving as the reference).

## 3 RESULTS

### 3.1 Results of intergroup analysis

#### 3.1.1 Univariate analysis

**FIGURE 2:**
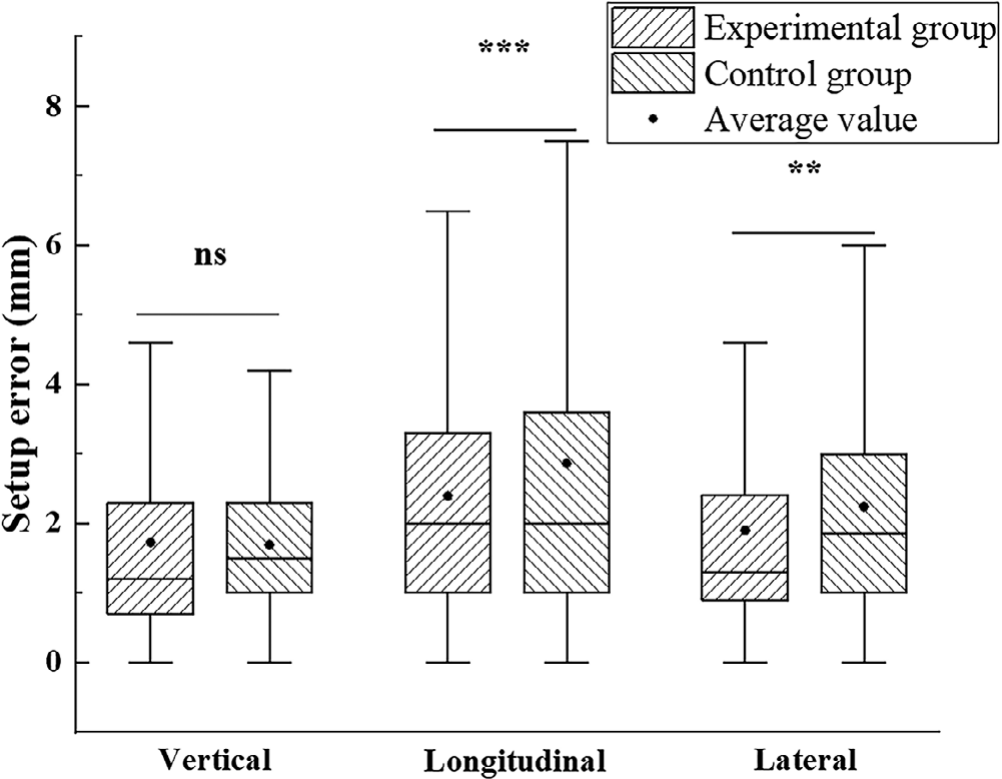
Box plot of setup errors in three directions for all patients between the experimental and control groups.

As shown in Table 3, for all included cases, the experimental group had median setup errors of 1.20 , 2.00 , and 1.30 mm in the vertical, longitudinal, and lateral directions, respectively. In contrast, the control group had median setup errors of 1.50 , 2.00 , and 1.85 mm in the same respective directions. The differences in setup errors between the two groups in the longitudinal and lateral directions were statistically significant (*p* < 0.05), as shown in Figure 2. For thorax and abdomen cases, the differences in setup errors between the two groups in the longitudinal and lateral directions were also statistically significant (*p* < 0.05). The setup differences for head and neck cases were observed in the vertical and longitudinal directions, while for limb cases, the differences were noted in the lateral direction. It is plain to see that, except for head and neck cases, the combination of styrofoam and thermoplastic mask significantly decreased setup errors in the longitudinal direction.

**TABLE 3:** Univariate analysis results of setup errors in the vertical, longitudinal, and lateral directions for different cases.

	Vertical (mm)			Longitudinal (mm)			Lateral (mm)		
Location	Experimental group	Control group	*p*	Experimental group	Control group	*p*	Experimental group	Control group	*p*
Total	1.73 ± 0.06	1.70 ± 0.05	0.09	2.40 ± 0.09	2.87 ± 0.11	<0.01*	1.90 ± 0.08	2.24 ± 0.08	<0.01*
Head and neck	1.51 ± 0.12	1.67 ± 0.08	0.02*	2.82 ± 0.21	2.10 ± 0.15	0.02*	1.53 ± 0.10	1.74 ± 0.14	0.76
Thorax	1.66 ± 0.12	1.62 ± 0.10	0.51	2.15 ± 0.14	3.10 ± 0.22	<0.01*	1.90 ± 0.13	2.55 ± 0.18	0.02*
Abdomen	1.76 ± 0.11	1.69 ± 0.08	0.55	2.53 ± 0.16	3.36 ± 0.20	<0.01*	1.73 ± 0.11	2.60 ± 0.17	<0.01*
Limb	2.02 ± 0.16	1.95 ± 0.15	0.68	2.02 ± 0.17	2.80 ± 0.40	0.15	1.95 ± 0.15	3.36 ± 0.46	<0.01*

^*^ *p* < 0.05.

#### 3.1.2 Multivariate analysis

The multivariate analysis results were generally consistent with the univariate analysis results, as shown in Table 4, 5, 6, 7, 8. In the vertical direction, the combination of styrofoam and thermoplastic mask showed no significant reduction in setup errors across different cases. BMI was the only factor significantly affecting setup errors, with higher BMI values being positively correlated with larger setup errors (*p* < 0.05). In the longitudinal direction, the multivariate analysis indicated a correlation between immobilization methods and setup error outcomes, except in limb cases. Notably, the experimental group exhibited larger setup errors in head and neck cases but smaller errors in thoracic and abdominal cases, aligning with the univariate analysis results. In the lateral direction, the multivariate analysis also showed that the use of styrofoam decreased setup errors. Other variables, such as surgical history and BMI, also significantly influenced setup errors.

**TABLE 4:** Multivariate analysis results in the vertical, longitudinal, and lateral directions for all cases.

	Vertical			Longitudinal			Lateral		
Variable	*β*	SD	*p*	*β*	SD	*p*	*β*	SD	*p*
Fixation device	−0.02	0.08	0.85	0.43	0.15	<0.01*	0.28	0.12	0.02*
Gender	−0.07	0.08	0.40	0.20	0.15	0.20	0.02	0.13	0.89
Age	<0.01	<0.01	0.84	<0.01	0.01	0.45	−0.01	<0.01	0.23
Educational level	0.10	0.08	0.19	−0.07	0.15	0.61	0.20	0.12	0.10
Surgical history	0.15	0.08	0.07	−0.14	0.15	0.35	0.47	0.12	<0.01*
BMI	0.03	0.01	0.02*	<0.01	0.02	0.94	0.03	0.02	0.06

Abbreviation: SD, standard deviation.

^*^ *p* < 0.05, *β* indicates unstandardized coefficient.

**TABLE 5:** Multivariate analysis results in the vertical, longitudinal, and lateral directions for head and neck cases.

	Vertical			Longitudinal			Lateral		
Variable	*β*	SD	*p*	*β*	SD	*p*	*β*	SD	*p*
Fixation device	0.14	0.14	0.31	−0.57	0.25	0.02*	0.21	0.18	0.24
Gender	0.12	0.15	0.45	0.68	0.27	0.01*	0.18	0.19	0.34
Age	0.01	0.01	0.14	−0.02	0.01	0.06	0.01	0.01	0.35
Educational level	−0.04	0.14	0.77	−0.33	0.26	0.20	0.17	0.18	0.33
Surgical history	0.13	0.15	0.36	0.53	0.26	0.04*	0.53	0.18	<0.01*
BMI	0.01	0.02	0.64	−0.12	0.03	<0.01*	0.01	0.02	0.60

Abbreviation: SD, standard deviation.

^*^ *p* < 0.05, *β* indicates unstandardized coefficient.

**TABLE 6:** Multivariate analysis results in the vertical, longitudinal, and lateral directions for thorax cases.

	Vertical			Longitudinal			Lateral		
Variable	*β*	SD	*p*	*β*	SD	*p*	*β*	SD	*p*
Fixation device	−0.16	0.18	0.38	0.91	0.29	0.02*	0.61	0.24	0.01*
Gender	−0.18	0.19	0.34	0.08	0.31	0.79	−0.25	0.26	0.33
Age	−0.02	0.01	0.08	−0.01	0.01	0.33	−0.05	0.01	<0.01
Educational level	0.06	0.16	0.71	−0.34	0.27	0.21	0.04	0.22	0.84
Surgical history	−0.13	0.18	0.48	−0.36	0.30	0.24	0.18	0.25	0.46
BMI	0.02	0.03	0.48	−0.07	0.05	0.15	−0.08	0.04	0.04*

Abbreviation: SD, standard deviation.

^*^ *p* < 0.05, *β* indicates unstandardized coefficient.

**TABLE 7:** Multivariate analysis results in the vertical, longitudinal, and lateral directions for abdomen cases.

	Vertical			Longitudinal			Lateral		
Variable	*β*	SD	*p*	*β*	SD	*p*	*β*	SD	*p*
Fixation device	−0.08	0.15	0.58	0.67	0.29	0.02*	0.84	0.22	<0.01*
Gender	−0.21	0.15	0.17	0.24	0.29	0.42	0.08	0.22	0.70
Age	0.01	0.01	0.02	−0.01	0.01	0.61	−0.01	0.01	0.28
Educational level	0.02	0.14	0.86	0.36	0.28	0.19	0.40	0.20	0.04*
Surgical history	0.08	0.14	0.55	−0.25	0.27	0.36	0.39	0.20	0.05*
BMI	0.02	0.02	0.26	0.15	0.04	<0.01*	0.14	0.03	<0.01*

Abbreviation: SD, standard deviation.

^*^ *p* < 0.05, *β* indicates unstandardized coefficient.

**TABLE 8:** Multivariate analysis results in the vertical, longitudinal, and lateral directions for limb cases.

	Vertical			Longitudinal			Lateral		
Variable	*β*	SD	*p*	*β*	SD	*p*	*β*	SD	*p*
Fixation device	−0.22	0.25	0.39	0.68	0.41	0.10	1.63	0.44	<0.01*
Gender	0.02	0.25	0.94	0.25	0.40	0.53	0.65	0.43	0.13
Age	<0.01	0.01	0.56	<0.01	0.01	0.81	0.02	0.01	0.09
Educational level	0.63	0.27	0.02*	0.19	0.44	0.67	0.01	0.47	0.97
Surgical history	0.46	0.30	0.13	−0.17	0.49	0.73	−0.20	0.53	0.71
BMI	0.06	0.03	0.06	−0.06	0.06	0.28	0.05	0.06	0.43

Abbreviation: SD, standard deviation.

^*^ *p* < 0.05, *β* indicates unstandardized coefficient.

#### 3.1.3 Anatomical deviations

**FIGURE 3:**
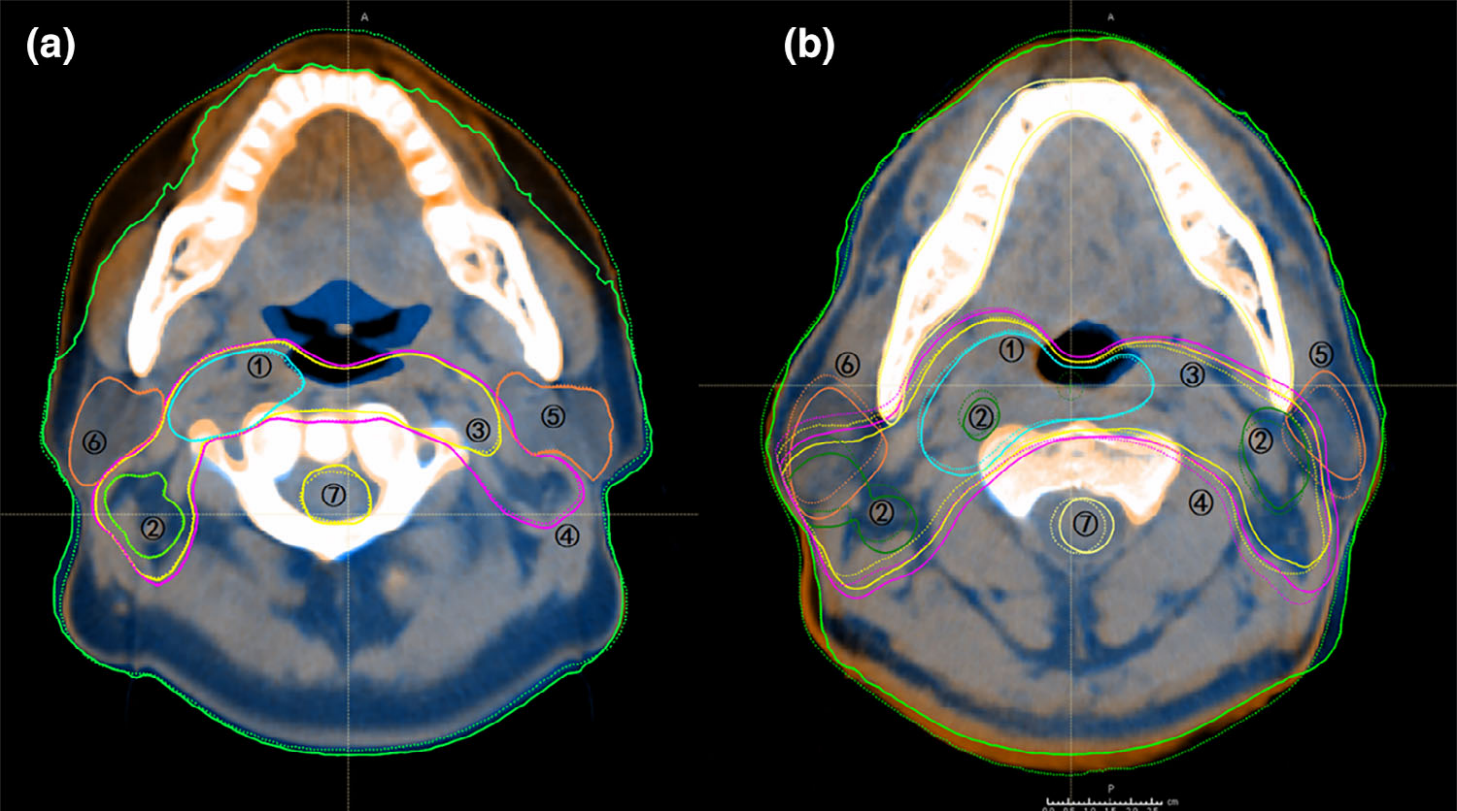
ROIs of planning CT (solid line) and CBCT (dotted line) in head and neck cases. (a) Control group, (b) experimental group. ①GTV‐nx, ②GTV‐nd, ③CTV1, ④CTV2, ⑤parotid gland (left), ⑥parotid gland (right), ⑦spinal cord.

**FIGURE 4:**
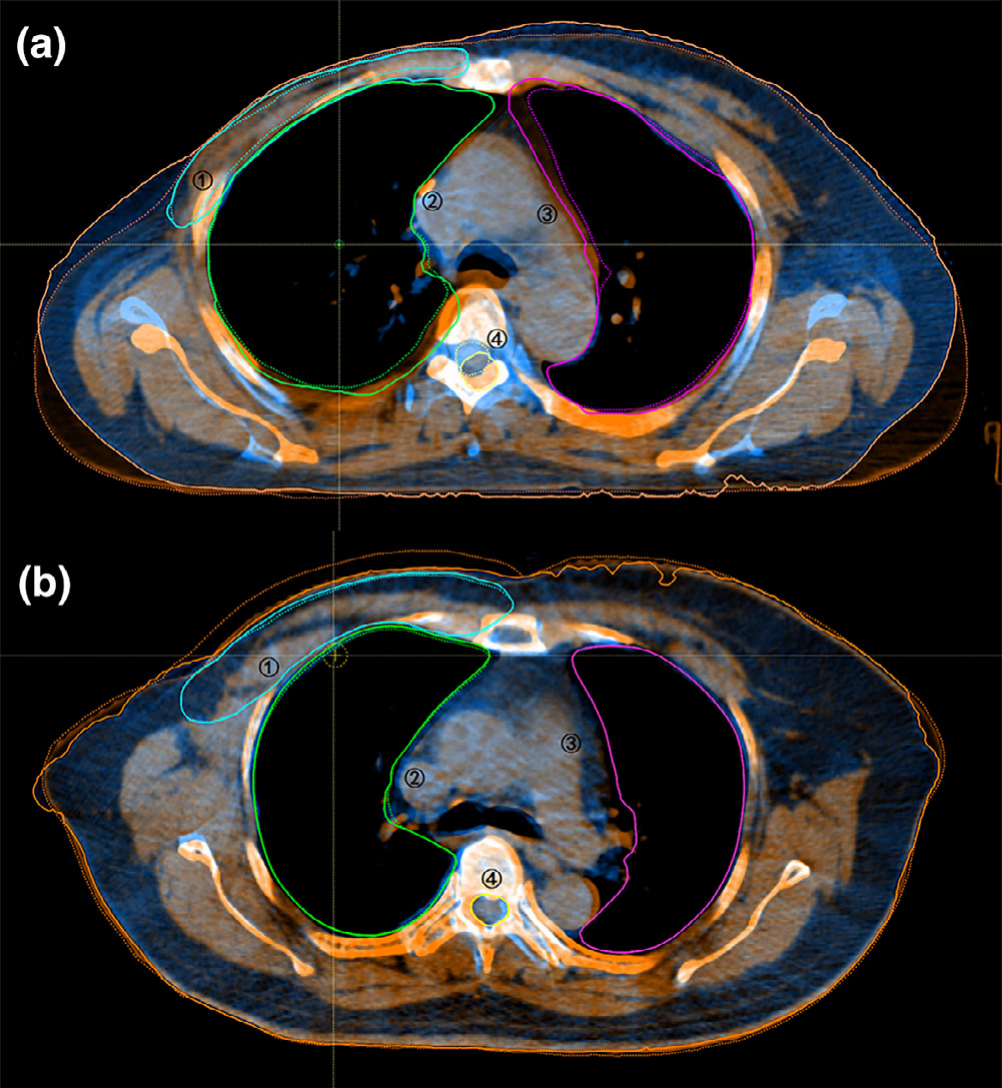
ROIs of planning CT (solid line) and CBCT (dotted line) in thoracic cases. (a) Control group, (b) experimental group. ①PTV, ②lung (right), ③lung (left), ④spinal cord.

**FIGURE 5:**
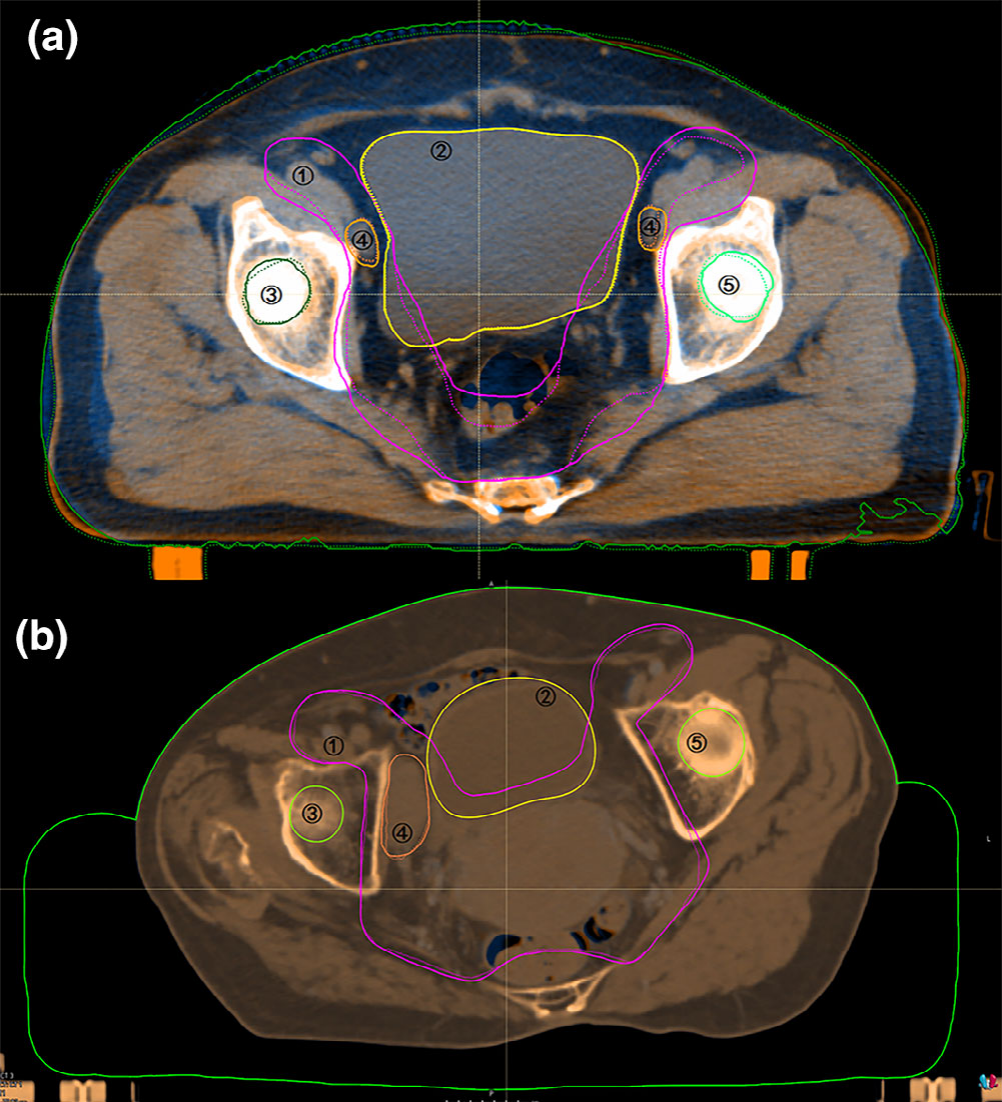
ROIs of planning CT (solid line) and CBCT (dotted line) in abdominal cases. (a) Control group, (b) experimental group. ①CTV, ②bladder, ③femoral head (right), ④GTV, ⑤femoral head (left).

Twenty head and neck, 20 thorax, and 20 abdomen cases were randomly selected from both the experimental and control groups, and the average DSC and ASSD values of ROIs are shown in Table 9. For head and neck cases, the control group demonstrated better overlap for targets and most organs at risk. The DSC and ASSD for the parotids were 0.97 and 0.64 mm in the control group, versus 0.83 and 2.25 mm in the experimental group. For thoracic cases, the experimental group's results were significantly better than the control group's. For abdominal cases, the two fixation devices performed similarly, except for the GTV and bladder. Table 9 also showed that small‐volume organs have lower DSC values than large‐volume organs. Figures 3, 4, 5 showed the image registration results between planning CT and CBCT. In thoracic and abdominal cases, styrofoam combined with thermoplastic mask significantly reduced changes in the shape and position of ROIs, while thermoplastic mask alone may cause soft tissue deformation. However, for head and neck cases, the experimental group's results showed larger errors than the control group's, consistent with Table 9.

**TABLE 9:** Average DSC and ASSD of ROIs between planning CT and CBCT in control and experimental groups.

	Control group		Experimental group	
ROIs	DSC	ASSD (mm)	DSC	ASSD (mm)
Head and neck				
GTV‐nx	0.87	1.47	0.79	2.17
GTV‐nd	0.84	1.18	0.70	2.05
CTV1	0.90	1.89	0.83	2.93
CTV2	0.95	2.43	0.88	3.73
Brain stem	0.95	0.93	0.89	1.49
Spinal cord	0.78	2.59	0.58	3.56
Lens	0.71	1.06	0.56	2.01
Optical nerves	0.84	1.10	0.75	1.65
Optical chiasm	0.81	1.10	0.72	1.98
Parotid glands	0.97	0.64	0.83	2.25
Thyroid	0.80	1.50	0.79	1.40
Thorax				
PTV	0.93	1.84	0.98	1.14
Lung‐L	0.80	12.77	0.97	3.32
Lung‐R	0.82	10.44	0.97	1.68
Spinal cord	0.34	9.02	0.84	2.44
Abdomen				
GTV	0.94	1.56	0.98	0.53
CTV	0.98	1.22	0.98	0.63
Intestine tract	0.98	1.47	0.99	0.78
Bladder	0.86	8.04	0.99	0.79
Femoral Head‐L	0.94	1.67	0.94	1.83
Femoral Head‐R	0.95	1.68	0.99	0.57

### 3.2 Results of intragroup analysis

Table 10 presents the results of the multivariate analysis for the experimental group, where most factors had no significant impact on setup errors across all three directions. Notably, in the longitudinal direction, setup errors for head and neck cases were greater than for other cases, contrasting with the other two directions. Table 11 shows the multivariate analysis results for the control group, indicating different factors influenced setup errors in different directions. In the vertical direction, age and BMI were risk factors for setup error, with females exhibiting smaller setup errors than males. In the longitudinal direction, thoracic, abdominal, and limb cases had greater setup errors than head and neck cases. In the lateral direction, age, BMI, and surgical history were all risk factors for setup error.

**TABLE 10:** Multivariate analysis results in the vertical, longitudinal, and lateral directions for the experimental group.

	Vertical			Longitudinal			Lateral		
Variable	*β*	SD	*p*	*β*	SD	*p*	*β*	SD	*p*
Gender	0.13	0.14	0.37	0.30	0.19	0.13	−0.24	0.19	0.20
Age	<0.01	<0.01	0.31	−0.01	0.01	0.25	−0.01	0.01	0.09
Educational level	0.17	0.13	0.19	−0.01	0.18	0.96	0.21	0.17	0.23
Surgical history	0.20	0.13	0.13	−0.11	0.18	0.54	0.26	0.18	0.15
BMI	0.04	0.02	0.07	−0.06	0.03	0.03*	0.02	0.03	0.51
Thorax	0.14	0.18	0.45	−0.67	0.25	<0.01*	0.84	0.24	<0.01*
Abdomen	0.27	0.17	0.13	−0.24	0.24	0.31	0.79	0.23	<0.01*
Limb	0.45	0.19	0.02*	−0.77	0.26	<0.01*	0.17	0.25	0.49

Abbreviation: SD, standard deviation.

^*^ *p* < 0.05, *β* indicates unstandardized coefficient.

**TABLE 11:** Multivariate analysis results in the vertical, longitudinal, and lateral directions for the control group.

	Vertical			Longitudinal			Lateral		
Variable	*β*	SD	*p*	*β*	SD	*p*	*β*	SD	*p*
Gender	−0.23	0.10	0.02*	0.13	0.24	0.60	0.33	0.17	0.05
Age	0.01	<0.01	<0.01*	<0.01	0.01	0.62	0.01	0.01	0.04*
Educational level	0.00	0.10	0.97	0.06	0.23	0.80	<0.01	0.16	0.99
Surgical history	0.07	0.10	0.52	0.03	0.25	0.90	0.35	0.18	0.04*
BMI	0.03	0.01	0.04*	0.04	0.03	0.16	0.07	0.02	<0.01*
Thorax	−0.05	0.13	0.67	0.98	0.31	<0.01*	0.34	0.22	0.12
Abdomen	−0.01	0.12	0.92	1.25	0.28	<0.01*	0.18	0.20	0.37
Limb	0.43	0.17	0.01*	0.80	0.40	0.04*	1.93	0.29	<0.01*

Abbreviation: SD, standard deviation.

^*^ *p* < 0.05, *β* indicates unstandardized coefficient.

## 4 DISCUSSION

Patient immobilization is fundamental to ensuring the accuracy and effectiveness of radiotherapy and plays a significant role in reducing patient movement and positional variations, as well as in maintaining patient comfort.^12^, ^13^, ^14^ Styrofoam, as a patient‐specific immobilization device, has been proven in numerous studies to be reliable and effective in patient immobilization. However, most of these studies have employed univariate analysis methods, neglecting the impact of other potential factors on setup errors. Furthermore, there are few reports on which patient populations are suitable for the use of styrofoam fixation device.

The univariate analysis results for head and neck cases indicated that the use of styrofoam primarily decreased setup error in the lateral direction, but paradoxically increased setup error in the longitudinal direction. Lin et al.’s study demonstrated that styrofoam immobilization technology offers superior localization accuracy in both the nasopharyngeal and neck regions compared to thermoplastic masks. However, they only quantified the three‐dimensional vector and did not separately analyze the setup errors in each of the three directions.^15^ In the study by Li et al., conclusions were observed that are in contrast to ours. Their results indicated that the setup error along the longitudinal axis was significantly smaller in the experimental group compared to the control group (*p* = 0.001), and there was no significant difference in setup errors between the two groups in the other directions.^16^ The most likely reason is that in our department, styrofoam immobilization is only used in patients with kyphosis, as the head thermoplastic mask with a headrest does not conform to patient's neck curvature.

There are several potential reasons for this phenomenon. First, patients with kyphosis often have a more pronounced forward tilt of the head and neck. This altered posture may not be adequately supported by the styrofoam and thermoplastic mask combination, leading to potential misalignments during treatment. Second, the styrofoam may not conform as closely to the unique curvature of the neck and shoulders in these patients, resulting in gaps or areas of uneven pressure. This can cause subtle but consistent shifts in the patient's position, particularly in the longitudinal direction, during treatment. Additionally, patients with kyphosis might suffer from discomfort, potentially resulting in slight movements or posture adjustments, thereby increasing setup errors. Previous studies have also highlighted that anatomical variations, especially in the cervical spine, can significantly influence setup reproducibility. For instance, Contesini et al.’s study found that for head and neck patients, setup errors are influenced by patient's physical features, with kyphosis increasing the setup errors by 65.4%.^17^ This suggests that the anatomical challenges posed by kyphosis may indeed have a substantial impact on the effectiveness of immobilization devices like styrofoam.

In fact, styrofoam immobilization was used for patients without kyphosis in thorax and abdomen cases, and it was clearly observed that the setup error in the longitudinal direction was smaller in the experimental group compared to the control group. This also suggested that styrofoam has decreased the setup error in the longitudinal direction for patients with normal spinal curvature, while kyphosis significantly affects the setup errors. Zhou et al. compared vacuum bags and styrofoam in 40 cases of breast cancer patients and found that the setup errors in the lateral, longitudinal, and vertical directions for the styrofoam group were 1.63 ± 1.29 , 1.46 ± 1.51 , and 1.30 ± 1.35 mm, respectively, all of which were smaller than those for the vacuum bag group.^18^ In our study, the setup errors in these three directions were 1.90 ± 0.13 , 2.15 ± 0.14 , and 1.66 ± 0.12 mm, which are close to theirs. Table 9 shows a higher overlap of ROIs between CT and CBCT in thoracic and abdominal cases after image registration, but a lower overlap in head and neck cases. This indicated that the styrofoam combined with thermoplastic mask not only effectively decreases setup errors but also better fixes soft tissues.

Although statistical significance was found between the experimental and control groups in some cases, most of the differences in setup errors are submillimetre in magnitude. However, we believe even minor improvements in setup precision can have meaningful clinical implications. First, for small volume tumors, submillimeter errors may change the irradiated volume, causing actual tumor doses to deviate from planned doses. This may leave part of the tumor in a low dose area, increasing recurrence risk. Second, in areas like the hippocampus and brainstem, where setup precision is critical, submillimeter errors can have serious consequences. For instance, in brain radiotherapy, setup errors might unnecessarily irradiate functional areas like the hippocampus, impacting cognitive function and quality of life. Additionally, submillimeter errors can also significantly alter dose distributions in radiotherapy techniques that require the minimum setup errors. For example, when performing stereotactic radiosurgery (SRS) using cones with diameters in the range of 4– 20 mm, a setup error of just 1 mm may lead to a significant change in the target dose.

The multivariate analysis results aligned with the univariate analysis results. There was no significant difference in setup errors between the two groups in the vertical direction, while the experimental group performed better in the longitudinal and lateral directions. For head and neck cases, gender, surgical history, and BMI influenced setup errors in the longitudinal direction. In thoracic cases, higher BMI was associated with smaller setup errors in the lateral direction. For abdominal cases, BMI also affected setup errors, and additionally, educational level and surgical history had a significant impact on setup errors in the lateral direction. In summary, surgical history and BMI have a considerable influence on setup errors, with postoperative patients exhibiting larger setup errors, which may be related to reduced mobility following surgery.

The intragroup analysis results indicated that factors such as age, gender, surgical history, and educational level had no significant impact on the setup errors in the experimental group. In contrast, for the control group, increased age and BMI were associated with larger setup errors. Additionally, gender and surgical history also influenced setup errors. Therefore, styrofoam is more suitable for a diverse population, while the use of thermoplastic masks necessitates consideration of the patient's physical condition.

The study's limitations include not considering specific tumor types, as we only analyzed the applicability of styrofoam for tumors in different locations. More importantly, in our study, all head and neck patients in the experimental group had kyphosis, while those in the control group did not. This non‐random allocation may have influenced the outcomes, particularly in the head and neck subgroup. Thus, the observed differences in setup errors between the two groups might be partially attributed to this selection bias, and the findings for the head and neck subgroup may not be generalizable to all head and neck patients. Future research should strive to employ more rigorous randomization methods to minimize these biases and more accurately evaluate the impact of styrofoam fixation across diverse patient populations.

## 5 CONCLUSION

The styrofoam group exhibited lower setup errors compared to the thermoplastic mask group for head and neck, thorax, abdomen, and limb cases, particularly in the longitudinal and lateral directions. The setup results by styrofoam fixation device were not significant affected by patient's physical conditions, such as gender, age, educational level, and surgical history. However, the tumor location has an impact on the setup errors. Based on these findings, we recommend considering styrofoam fixation devices for patients with tumors in the thorax and abdomen, as it can effectively reduce setup errors and improve treatment accuracy. For head and neck patients with normal spinal curvature, styrofoam can also be a suitable option. However, styrofoam may not be appropriate for head and neck patients with kyphosis, as it might increase longitudinal setup errors in such cases.

## AUTHOR CONTRIBUTIONS

Study concept/study design: Jun Chen, FeiBao Guo; statistical analysis and manuscript writing: Yi Guo, Jun Chen, FeiBao Guo; data acquisition: Changsheng Wang, Simin Lin, Linglong Shao, Linzhen Lan, Jun Chen.

## CONFLICT OF INTEREST STATEMENT

No conflicts of interest.

## ETHICS STATEMENT

Not applicable.

## Data Availability

Not applicable.
